# Phyllotactic regularity requires the Paf1 complex in *Arabidopsis*

**DOI:** 10.1242/dev.154369

**Published:** 2017-12-01

**Authors:** Kateryna Fal, Mengying Liu, Assem Duisembekova, Yassin Refahi, Elizabeth S. Haswell, Olivier Hamant

**Affiliations:** 1 Laboratoire Reproduction et Développement des Plantes, Univ Lyon, ENS de Lyon, UCB Lyon 1, CNRS, INRA, F-69342, Lyon, France; 2 Sainsbury Laboratory, University of Cambridge, Bateman Street, Cambridge CB2 1LR, UK; 3 Department of Biology, Mailbox 1137, Washington University in Saint Louis, Saint Louis, MO 63130, USA

**Keywords:** Phyllotaxis, Meristem, Paf1c, Auxin, Variability, Reproducibility

## Abstract

In plants, aerial organs are initiated at stereotyped intervals, both spatially (every 137° in a pattern called phyllotaxis) and temporally (at prescribed time intervals called plastochrons). To investigate the molecular basis of such regularity, mutants with altered architecture have been isolated. However, most of them only exhibit plastochron defects and/or produce a new, albeit equally reproducible, phyllotactic pattern. This leaves open the question of a molecular control of phyllotaxis regularity. Here, we show that phyllotaxis regularity depends on the function of VIP proteins, components of the RNA polymerase II-associated factor 1 complex (Paf1c). Divergence angles between successive organs along the stem exhibited increased variance in *vip3-1* and *vip3-2* compared with the wild type, in two different growth conditions. Similar results were obtained with the weak *vip3-6* allele and in *vip6*, a mutant for another Paf1c subunit. Mathematical analysis confirmed that these defects could not be explained solely by plastochron defects. Instead, increased variance in phyllotaxis in *vip3* was observed at the meristem and related to defects in spatial patterns of auxin activity. Thus, the regularity of spatial, auxin-dependent, patterning at the meristem requires Paf1c.

## Abstract (precis)

**Summary:** Analysis of divergence angles in VIP mutants reveals that the regularity of organ initiation at the shoot apical meristem requires Paf1c and is related to spatial patterns of auxin activity.

## INTRODUCTION

‘*Dreams apart, numerical precision is the very soul of science, and its attainment affords the best, perhaps, the only criterion of the truth of theories and the correctness of experiments*’ (p. 2, Thompson, 1942).

Following in the footsteps of D'Arcy Thompson's *On Growth and Form*, developmental biology is becoming increasingly quantitative. With the accumulation of fine-grained quantitative data, inherent variability in development is currently emerging as an instructional cue. Conversely, the analysis of mutants with excessive or reduced variability in particular features helps us unravel how developmental reproducibility arises (Singh et al., 2010; Wernet et al., 2006; Laslo et al., 2006; Gupta et al., 2011; Uyttewaal et al., 2012; Hong et al., 2016; Abley et al., 2016).

The pattern of organ position along the plant stem has fascinated scientists for centuries because of its stereotyped regularity. Both spatial and temporal factors contribute to the final architecture of a shoot. Aerial organs are initiated from the shoot apical meristem (SAM), where plant stem cells are located, and are called primordia at that stage. In *Arabidopsis*, successive primordia emerge from the SAM in a Fibonacci spiral, with a new primordium emerging every 137°. This angle between adjacent primordia is called the divergence angle, and is very regular in wild-type (WT) plants. In the strictest sense, phyllotaxis refers to this spatial meristematic pattern only. As stem growth occurs, the initial phyllotaxis pattern does not necessarily lead to an equally regular pattern along the stem. There are two main reasons for this. First, because successive organs are separated by an internode, stem twisting can increase or decrease the angle between successive organs as the stem grows (Landrein et al., 2013). Second, the plastochron, i.e. the time between the emergence of successive primordia, can affect the final divergence angles along the stem. Indeed, if the average plastochron is very small, it can locally reach negative values, and, as organs are initiated before internode grows, a permutation in the sequence of organ emergence is possible. How could this lead to an altered architecture in the end? Let's take the example of a simple permutation: primordium 3 would emerge before primordium 2 at the meristem; later on, whereas organ 1 would originate from primordium 1, the contiguous organ along the stem would originate from primordium 3 instead of primordium 2, thus leading to a divergence angle of 137+137=274°. Such permutations are widespread and can be observed in WT *Arabidopsis* (Besnard et al., 2014; Landrein et al., 2015b; Guédon et al., 2013; Refahi et al., 2011).

Recently, the question of phyllotactic variability has emerged (Mirabet et al., 2012; Refahi et al., 2016; Besnard et al., 2014; Landrein et al., 2015b). However, all experimental work on the regularity of phyllotactic patterns in fact refers to reproducibility of plant architecture. In particular, none of the mutants with variable architecture exhibit a variable spatial pattern of organ initiation at the SAM. This is notably the case for mutants with defects in the plastochron, leading to permutations in the final organ positions along the stem: despite the increased variance in divergence angle between fully differentiated organs, primordia are still initiated along the stereotypical 137° divergence angle at the meristem (e.g. Couder, 1998; Mirabet et al., 2012; Besnard et al., 2014; Landrein et al., 2015b; Guédon et al., 2013). Genetic factors have been implicated in the control of phyllotactic modes, with mutants switching between spiral and alternate patterns for instance (Giulini et al., 2004; Prasad et al., 2011), but not in the maintenance of phyllotactic regularity.

Whereas increased variability in leaf positions has been reported in the rice mutants for the *SHO1*, *2* and *3* homeobox genes, qualitative observation of shoot meristems indicates that these defects might be correlated with variability in meristem shape rather than in phyllotaxis (Itoh et al., 2000). Aberrant architecture is also observed in *clavata3* mutants, which exhibit meristems up to 1000 times bigger than the WT (Fletcher et al., 1999; Szczesny et al., 2009; Leyser and Furner, 1992). However, the extent to which morphogenetic defects in such mutant meristems are directly related to phyllotaxis is not yet clear. When meristems are larger, the average plastochron is also smaller and permutations between successive organs are more frequent, even in the WT (Landrein et al., 2015b), hampering the analysis of phyllotaxis regularity at the meristem. Therefore, mutants with normal or smaller meristems would be more useful for investigating the question of the control of phyllotactic variance at the meristem.

Auxin has been indirectly implicated in phyllotaxis both through genetics and through modeling approaches. *PIN1* encodes an auxin efflux carrier and is required to generate discrete auxin peaks where organs will later emerge in the SAM (Reinhardt et al., 2003). MP, an auxin response factor, was recently shown to control the polarity of the auxin efflux carrier PIN1, in a positive-feedback loop, reinforcing the spatial patterns of auxin peaks at the shoot meristem (Bhatia et al., 2016). Defective organogenesis has been recorded in severely affected mutants, such as *pin-formed 1* (*pin1*; Gälweiler et al., 1998) or *monopteros* (*mp*; Aida et al., 2002), in which no organs are generated. Although these important observations consolidate a role of auxin transport and transduction in organ initiation, and their periodic emergence, they do not formally demonstrate a role of auxin in the spatial regularity of phyllotaxis, notably because of the severity of the mutant phenotypes.

Computational simulations of auxin transport in the meristem suggest that a stable phyllotactic pattern can emerge from the local response to auxin flow or concentration, or a combination of both (Reinhardt et al., 2003; Heisler et al., 2005; de Reuille et al., 2006; Smith et al., 2006; Bayer et al., 2009; Heisler et al., 2010; Stoma et al., 2008; Sahlin et al., 2009). However, these theoretical results do not formally demonstrate that auxin is sufficient to generate stable patterns, notably because mutants with increased phyllotactic variance have not been isolated so far. Other models, involving geometrical features (namely the size of the stem cell niche in the meristem and the size of fields inhibiting outgrowth around emerging primordia) further show how phyllotaxis can, in principle, stably self-maintain or switch between equally reproducible patterns (Douady and Couder, 1992). We are thus left with a picture in which phyllotactic regularity emerges because it is heavily constrained, by geometry or auxin transport, leaving the question of a possible molecular control of phyllotactic variance unanswered.

The RNA polymerase-associated factor 1 complex (Paf1c) plays a role in transcription-related processes such as the facilitation of elongation, recruitment of chromatin remodeling factors (histone methylation) and polyadenylation in yeast, plants and animals (Koch et al., 1999; Nordick et al., 2008; Penheiter et al., 2005; Sheldon et al., 2005; Sadeghi et al., 2015; Jaehning, 2010; Dermody and Buratowski, 2010; Chu et al., 2013; Oh et al., 2008). Plant Paf1c contains several subunits that are functionally homologous to those present in animal cells, including VERNALIZATION INDEPENDENCE (VIP) 3, VIP4, VIP5, EARLY FLOWERING (ELF) 7, VIP6 (also known as ELF8) and PLANT HOMOLOGOUS TO PARAFIBROMIN (PHP) (He et al., 2004; Oh et al., 2004; Jaehning, 2010; Oh et al., 2008; Park et al., 2010; Zhang and van Nocker, 2002). Consistent with VIP3, VIP4, VIP5 and VIP6 contributing to a plant Paf1c homolog, these proteins co-immunoprecipitate (Oh et al., 2004) and the corresponding single mutants display similar growth defects, which include reduced plant size, severely affected fertility and early flowering (Zhang et al., 2003; Takagi and Ueguchi, 2012; Dorcey et al., 2012). Recently, a role for Paf1c in patterning has emerged in animals, notably in cell lineage specification (Akanuma et al., 2007; Nguyen et al., 2010; Langenbacher et al., 2011; Kim et al., 2012; Zhang et al., 2013; Kubota et al., 2014). Here, we provide evidence that the regularity of the spatial pattern of auxin activity and organ initiation at the SAM requires Paf1c.

## RESULTS

### Paf1c mutants display cotyledon number defects

**Fig. 1.**
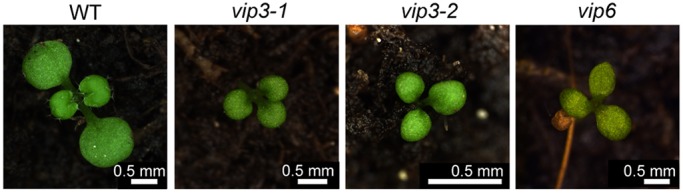
***vip* mutants exhibit a higher number of seedlings with three cotyledons.** Representative 8-day-old WT (*Col-0*) plants and tricots found in *vip3-1*, *vip3-2* and *vip6*.

As reported previously, we observed that *vip3* mutants exhibit growth defects (Zhang et al., 2003; Takagi and Ueguchi, 2012; Dorcey et al., 2012). However, we also noticed that *vip3* seedlings frequently exhibit three cotyledons instead of two. Approximately 9% of *vip3-2* (112/1235; *n*=8 independent populations; T-DNA insertion in the first exon of *VIP3*) and nearly 2% of *vip3-1* (14/766; *n*=7 independent populations; T-DNA insertion in the 2nd exon of *VIP3*) 8-day-old seedlings displayed an altered cotyledon number, compared with a very low frequency in the WT (Fig. 1; no tricotyledons were observed in 1742 plants examined; *n*=8 independent populations). Similar cotyledon number defects were observed for seedlings deficient in VIP6, another component of the *Arabidopsis* Paf1 complex (Oh et al., 2004): around 6% of *vip6* 8-day-old seedlings displayed three cotyledons (Fig. 1; 52/805; *n*=8 independent populations). Note that the presence of tricot seedlings was previously described for another allele of *vip3* (*bouquet-1* allele); however, it was thought to be associated with the dominant-negative effect of the point mutation in this allele (Takagi and Ueguchi, 2012). Such defects had also been observed in several mutants affected in organogenesis and auxin distribution, such as *pinoid* or *pin1*, prompting us to search for other architectural defects in the *vip3* and *vip6* mutants.

### Paf1c mutants display architecture defects

**Fig. 2.**
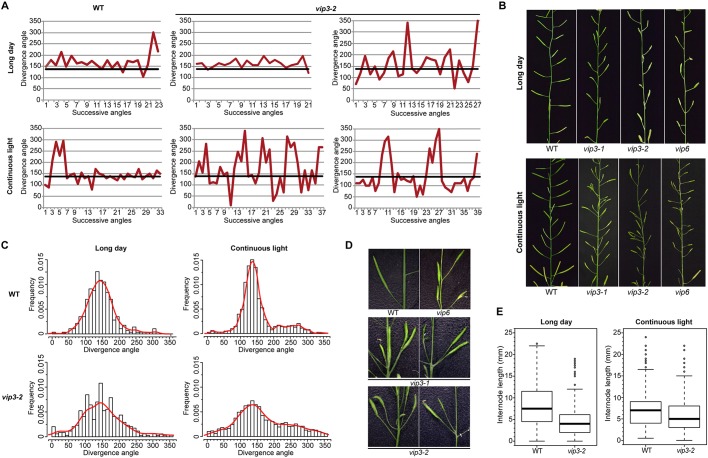
**Architecture is affected in *vip3* and *vip6* mutant plants.** (A) Sequence of divergence angles along the inflorescence stem in a representative WT plant and two representative *vip3-2* mutant plants grown in long-day conditions at 21°C (upper panel, ‘long day’) or short days at 21°C for 3 weeks followed by continuous light at 16°C (lower panel, ‘continuous light’). The thick black line on each graph corresponds to the canonical angle of 137°. (B) Images of inflorescence stems in WT and *vip* mutant plants. (C) Frequency of divergence angles between successive siliques in WT and *vip3-2* plants grown in long-day (left; WT: 305 angles, 20 plants; *vip3-2*: 343 angles, 15 plants) and continuous light (right; WT: 1997 angles, 54 plants; *vip3-2*: 1767 angles, 52 plants) conditions. (D) Close-ups of inflorescence stems of WT and *vip* mutants, illustrating the more frequent perturbations in the sequence of organ initiation and colocalized siliques in *vip* mutants. (E) Boxplots displaying the average internode length on the stems of WT and *vip3-2* plants grown in long-day (left; WT: 520 internodes, 20 plants; *vip3-2*: 420 internodes, 21 plants) and continuous light (right; WT: 620 internodes, 21 plants; *vip3-2*: 677 angles, 18 plants) conditions. The black line in the boxplot represents the median, the box represents the distribution range of 50% of the measured values and the bars (whiskers) illustrate the upper and lower quartiles (25% of the measured values that fall outside of the inter-quartile range).

As previously reported (e.g. Zhang et al., 2003), *vip3* mutant plants display a semi-dwarf phenotype and strong male sterility when grown in long-day conditions (i.e. 16 h light/8 h dark, 21°C). Under these conditions, both the average number of siliques and the stem length decreased in *vip* mutants compared with WT plants (Fig. S1). Qualitatively, we observed aberrant angles between successive siliques along the stem, but these can also, though rarely, be observed in the WT (Fig. 2B,D). As described above, in principle, these defects could be caused by stem twisting, changes in phyllotaxis, or changes in plastochron.

Changes in plastochron are likely to contribute to architecture defects in *vip* mutants. Irregular timing of organ outgrowth is frequent even in the WT, leading to permutations between the final positions of successive organs, generating detectable signatures in divergence angle sequences along the stem (Besnard et al., 2014; Landrein et al., 2015b): typically, a 274°-223°-274° sequence between successive organs along the stem instead of 137°-137°-137° marks one permutation event involving two organs (Guédon et al., 2013; Refahi et al., 2011). Depending on the number of organs involved in a permutation and the chaining of such events, the distribution of divergence angles in a population of plants with plastochron defects will show a characteristic set of peaks centered around multiples of the canonical angle 137° (Guédon et al., 2013; Refahi et al., 2011). For instance, in the WS-4 ecotype, the presence of a larger meristem is associated with more frequent organ permutations, leading to new peaks around 274° (2α), 223°(−α) and 51°(3α) and thus a reduced peak at 137° (α) (Landrein et al., 2015b).

To determine whether changes in plastochron could explain the observed phenotype in *vip3*, we used a 3D protractor (Peaucelle et al., 2007) to report divergence angles in WT and *vip3-2*, grown in long-day conditions (WT: 304 angles measured from 15 plants; *vip3-2*: 343 angles measured from 21 plants; Fig. 2A). Although the average divergence angle was close to 137° in *vip3-2*, as in the WT, the distribution of angles in the *vip3-2* was flatter and wider. The presence of secondary peaks around 275° and 223° could reflect the presence of organ permutations (Fig. 2C) (Guédon et al., 2013; Besnard et al., 2014; Landrein et al., 2015b), but these angle signatures were not prominent, suggesting that defects in plastochron might not be sufficient to explain the *vip3* phenotype.

We next analyzed every divergence angle sequence individually using the same mathematical method as described by Besnard et al. (2014) to test whether the increased variability in the *vip3-2* divergence angles could be entirely explained by two-organ or more complex permutations. We found that the number of permutations involving two or three organs was higher in *vip3-2* than in the WT (17% and 3.3% of permutated organs, respectively) (Table 1). However, the presence of permutations involving up to five organs did not fully explain the observed variability in *vip3-2* angle distribution: 22% of the *vip3-2* divergence angle sequences could not be explained by plastochron defects and associated organ permutations, compared with 3.5% in the WT (see Table 1). To confirm this conclusion, we performed the same analysis in *vip3-1* and in *vip6* and we obtained similar results, with up to 74% of angles in *vip6* unexplained by plastochron defects (*vip3-1*: 201 angles, 15 plants; *vip6*: 360 angles, 17 plants; Fig. S2, Table 1). Thus, these data suggest that the aberrant divergence angles found on *vip3* and *vip6* mutant stems are caused by defects both in the temporal sequence of organ initiation (plastochron) and in the spatial pattern of organ initiation at the meristem (phyllotaxis).

**Table 1.**
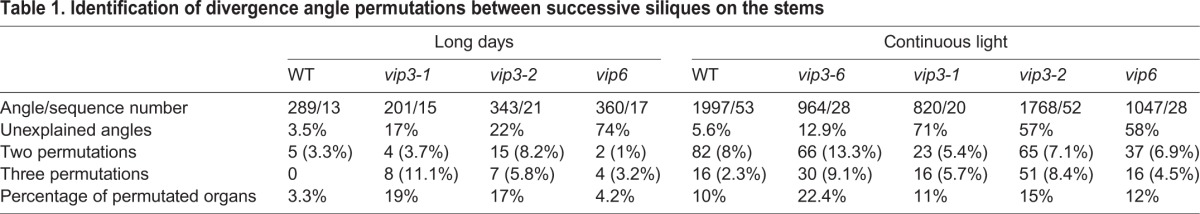
**Identification of divergence angle permutations between successive siliques on the stems**

### Continuous light and low temperature partially restore growth and enhance patterning defects in Paf1c mutants

To test whether aberrant divergence angles can be detected in *vip3* and *vip6* mutant meristems, organ initiation patterns were analyzed by meristem dissection. Although the previously used long-day conditions are the most commonly used conditions in *Arabidopsis* research worldwide, these conditions were not appropriate to pursue our analysis of *vip3* and *vip6* mutants, as apices were so small and fragile that dissection was close to impossible. We thus tested new growth conditions in which plants could be more vigorous. This also allowed us to test whether the increased variance in divergence angle remained when growth conditions were more favorable to stem growth.

In line with a previous report that the floral phenotype of *vip3* plants can be attenuated by lower growth temperature (Zhang et al., 2003), we grew *vip3-2* plants for 3 weeks in short-day conditions at 21°C, and then transferred them to continuous light at 16°C (this scheme is referred as ‘continuous-light conditions’ thereafter). Under these conditions, WT plants and their apices were bigger, and stem length as well as average silique number in *vip3-1*, *vip3-2* and *vip6* mutants were closer to the WT than were those of plants grown in long days (Fig. S1). The average stem internode length of *vip3-2* mutant plants was not significantly different from that of WT plants, but the frequency of shorter internodes remained slightly higher in *vip3-2* (Fig. 2E).

We next quantified the plastochron defects in these plants, expecting that restored growth might produce patterns in *vip3-2* closer to canonical patterns (WT: 1997 angles, 54 plants; *vip3-2*: 1768 angles, 52 plants). Instead, *vip3-2* mutants displayed similar architecture defects in continuous-light conditions and in long days. In WT plants, the expected secondary peaks at approximately 223° and 274° could be detected more clearly in continuous light than in long days, but these peaks remained relatively small in *vip3-2* (Fig. 2C).

To quantify the number of permutations in these angle sequences, we performed the same mathematical analysis as above. We found that continuous-light conditions promoted permutations involving two or three organs in both the WT and *vip3-2* mutants (Table 1). These growth conditions also increased the proportion of unexplained angles in *vip3-2* mutants; 57% of the angles could not be explained by permutations (compared with 6% in the WT, Table 1). To confirm this observation, we performed the same analysis in the *vip3-1* allele and in *vip6* and made similar observations (*vip3-1*: 820 angles, 20 plants; *vip6*: 1047 angles, 28 plants; Fig. 2B, Fig. S2, Table 1).

Because *vip3-1*, *vip3-2* and *vip6* mutants exhibit strong growth defects, we also analyzed the divergence angles between successive siliques in the recently reported *vip3-6* allele (measuring 964 angles from 28 plants), which displays a very weak growth phenotype (Jensen et al., 2017). In this background, we still detected an increase in the variability of divergence angles along the stem, and 12.9% of these angles could not be explained by plastochron defects (Table 1).

Taken together, these data strongly suggest that architecture defects in Paf1c mutants are not only the result of plastochron defects (i.e. in the temporal sequence of organ emergence), but are also caused by defective spatial patterns of organ initiation at the SAM. So far, the only known mutants with such quantified defects are *clasp-1* (with an increase in 100° divergence angle at the meristem, probably because of reduced meristem size; Landrein et al., 2015b), *abphyl* in maize and *plethora* in *Arabidopsis*, in which a switch in phyllotactic mode is observed (Giulini et al., 2004; Prasad et al., 2011). Interestingly, none of these mutants exhibits a variable phyllotactic pattern, but instead exhibit a reproducible, albeit different, phyllotactic mode. Given the observed divergence angle distribution along the stem, *vip3* and *vip6* mutant alleles might thus be specifically affected in phyllotactic regularity at the meristem.

### *vip3* mutants exhibit small meristems and altered *MONOPTEROS* expression

**Fig. 3.**
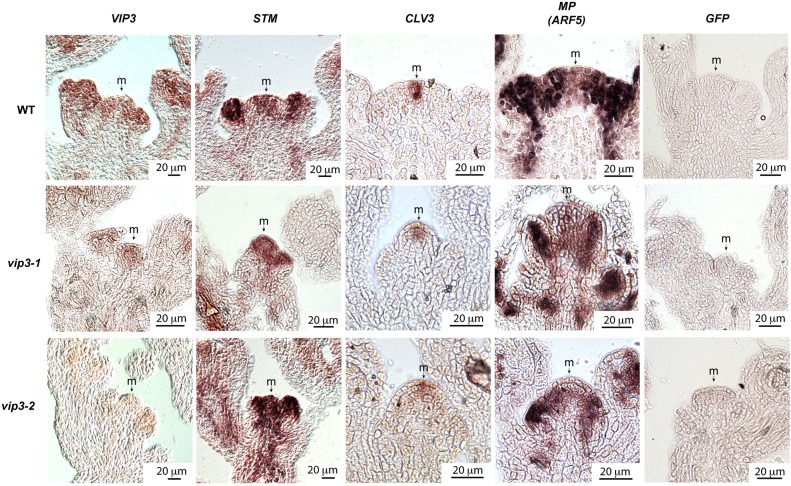
***VIP3* is expressed in the SAM, and its absence primarily affects the peripheral zone.** *In situ* hybridization of *VIP3*, *STM*, *CLV3* and *MP* transcripts in the shoot apex of WT and *vip3* (*vip3-1* and *vip3-2*) plants grown in short-day then continuous-light (16°C) conditions (as previously described). The antisense probe for *GFP* was used as a negative control. m, meristem.

To investigate the role of *VIP3* in patterning organ initiation at the SAM, we first checked whether *VIP3* is expressed in this tissue at the inflorescence stage. *VIP3* was previously reported to be ubiquitously expressed in *Arabidopsis* tissues (Zhang et al., 2003) and its expression was detected in the shoot apex 10 days after germination (Takagi and Ueguchi, 2012). Here, we confirmed by *in situ* hybridization that *VIP3* mRNA is indeed enriched in the inflorescence meristem (Fig. 3).

**Fig. 4.**
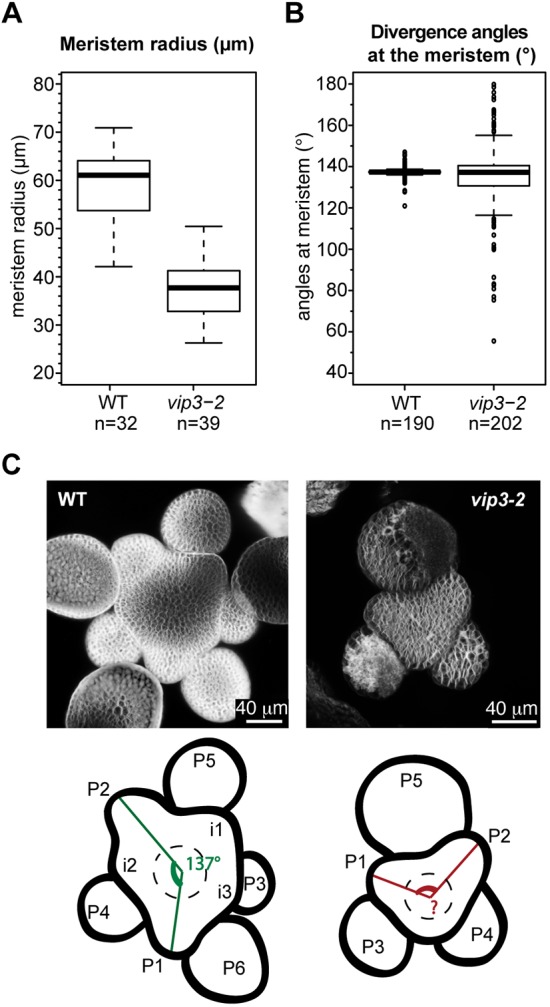
**Phyllotaxis is more variable in *vip3* mutant shoot meristems.** (A) Meristem radius (µm) in WT (*n*=32) and *vip3-2* (*n*=39) plants, grown in short-day conditions at 21°C for 3 weeks, then continuous-light conditions at 16°C. Meristem size was measured as described by Landrein et al. (2015b); α≤0.05% by two-tailed Student's *t*-tests. (B) Divergence angles between successive primordia in WT (*n*=190) and *vip3-2* (*n*=202) mutant meristems. The boxplots represent the median and the quartile range distribution of the measured values. (C) Top: representative WT and *vip3-2* meristems labeled with FM4-64. Bottom: schematics of the WT and *vip3-2* meristems displayed above, with the highlighted angles between the organs; initia are numbered from oldest to youngest (i1 to i3) and primordia are numbered from youngest to oldest (P1-P6).

Consistent with its expression pattern, VIP3 also controls meristem size: we found that *vip3* and *vip6* mutant meristems are about half the size of WT meristems (*r*≈38 µm in *vip3-2*, *r*≈34 µm in *vip3-1*, *r*≈36 µm in *vip6* versus *r*≈61 µm for WT and *vip3-6*; Fig. 4A, Fig. S3B). However, altered meristem size does not necessarily lead to phyllotactic defects: in WT plants, different growth conditions produce different meristem sizes, yet a stereotypical spatial pattern of organ initiation is conserved. In fact, reduction in meristem size has been associated with an increased plastochron (because inhibitory fields around each organ would encompass a proportionally larger domain when meristems are smaller), leading to a more clear-cut separation between successive primordia, reduced number of organ permutations and thus a more regular architecture (Landrein et al., 2015b). Consistent with these observations, the *CLV3*- and *WUS*-expressing zones of the meristem have recently been shown to scale to meristem size through a geometrical feedback involving cytokinin diffusion from the meristem L1 layer (Gruel et al., 2016), consistent with the maintenance of phyllotaxis regularity in smaller meristems. To check whether such scaling occurs in *vip3* mutants, we next analyzed the expression of *STM* (a whole meristem marker; Long et al., 1996), *CLV3* (a central zone marker; Fletcher et al., 1999) and *MONOPTEROS* (*MP*; also known as *ARF5*) (a peripheral zone marker; Rademacher et al., 2011) (Fig. 3). A genetic interaction had previously been reported for *VIP3* and *STM* (Takagi and Ueguchi, 2012). Using *in situ* hybridization, we found that the expression pattern of *STM* was not qualitatively different in *vip3* than in the WT, and confirmed that the domain with meristem identity, marked with *STM* signal, is smaller in *vip3* shoot apices. Surprisingly, we observed no major modification in the size of the *CLV3* expression domain, even though meristems were half the size of the WT (as shown above). Thus, the reduction in size is probably due to a reduction in the size of the peripheral zone. In contrast, the pattern for *MP* mRNA, a peripheral zone marker encoding an auxin response factor, appeared to be affected in *vip3* SAMs, with a marked reduction of *MP* signal in *vip3* meristem (Fig. 3). Using a *pMP::3xGFP* reporter line, we confirmed that *MP* expression is reduced in the meristem, consistent with our *MP in situ* hybridization results (Fig. S4D).

### *vip3* exhibits increased phyllotactic variance at the SAM

We next measured the divergence angles between successive primordia in *vip3-1*, *vip3-2*, *vip3-6*, *vip6* and WT meristems from plants grown in continuous-light conditions, as described by Besnard et al. (2014). We found divergence angles in the WT to be at 137±2.8°, consistent with previous reports (Fig. 4B,C) (Besnard et al., 2014; Landrein et al., 2015b, 2013). Although the average divergence angle was also around 137° in *vip3* and *vip6* mutants, the variance was significantly increased (Fig. 4B,C; *vip3-6*: 137±9.2° *vip3-1*: 137±11°, *vip3-2*: 136±17°, *vip6*: 136±14°). Furthermore, the number of angle outliers was much higher in *vip3* and *vip6* mutants than in the WT, in which divergence angles very rarely deviated from the canonical angle. The *vip3* and *vip6* mutants are also different from mutants affected in plastochron such as *ahp6*, which exhibit no defect in the variance of divergence angle at the meristem like the WT (Besnard et al., 2014), consistent with a specific defect in phyllotaxis in *vip3* and *vip6*.

**Fig. 5.**
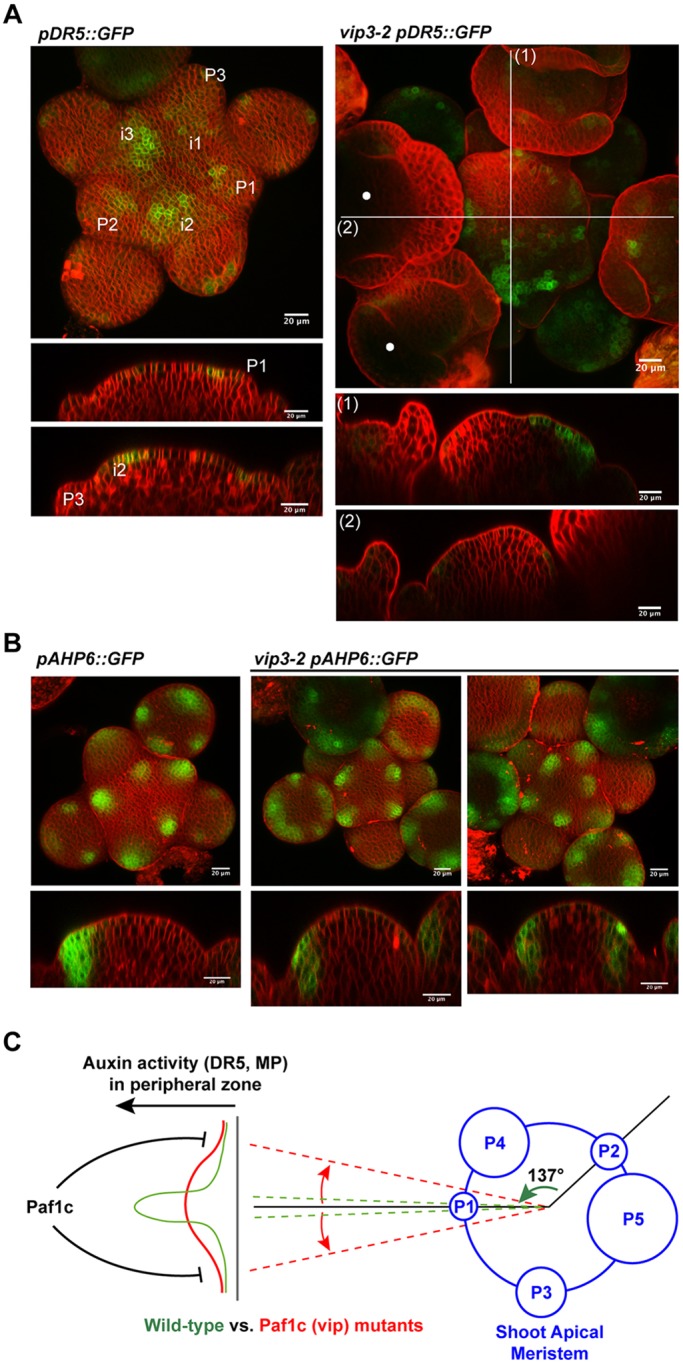
***pDR5::GFP* and *pAHP6::GFP* expression in *vip3* meristems.** (A) Surface projections and orthogonal sections of *pDR5::GFP*-expressing meristems in WT (left) and *vip3-2* (right) labeled with FM4-64. *vip3-2* meristem shows a perturbed phyllotaxis and abnormal *pDR5::GFP* expression pattern compared with the WT. In this case, phyllotaxis cannot be easily deduced from morphology or *pDR5::GFP* pattern (see Fig. S4A for other examples). The white dots indicate young flowers of similar age next to each other. White lines in top-right image (1,2) indicate the cross-sections shown below. (B) Surface projections and orthogonal sections of *pAHP6::GFP*-expressing meristems in WT (left) and *vip3-2* (right) labeled with FM4-64. Note the presence of AHP6-expressing emerging organs in *vip3*, with more (left) or less (right) abnormal divergence angles. Scale bars: 20 μm. (C) Members of the Paf1 complex (VIP3 and VIP6) are required to channel auxin activity in the peripheral zone of the meristem, and thus contribute to the spatial regularity of phyllotaxis. P, primordia.

As the initiation of new organs at the SAM is associated with increased auxin content and activity, the *pDR5::GFP* (*DR5*) auxin activity reporter line (Ulmasov et al., 1997) was introgressed in *vip3-2* and its pattern of expression analyzed by confocal microscopy. In WT meristems, we observed peaks of *DR5* expression marking the early pattern of organ initiation, as previously reported (Fig. 5A) (e.g. Smith et al., 2006; Heisler et al., 2005; Vernoux et al., 2011). In *vip3-2* meristems, *DR5* expression peaks were detected at the sites of incipient primordia but signal was also present outside these regions, and sometimes close to the central zone (Fig. 5A, Fig. S4A; *n*=30 for *vip3-2*, *n*=18 for WT). The presence of abnormal organ positions in the SAM could also be confirmed in this genetic background, with the occasional presence of adjacent organs at similar age (Fig. 5A, white dots). Together with the altered pattern of MP expression in *vip3*, these data demonstrate that the pattern of auxin activity is disturbed in *vip3* mutant apices, indicating that *VIP3* is required for the spatial regularity of auxin peaks, and thus of organ initiation, at the SAM (Fig. 5C). This also suggests that self-organizing processes are not robust enough to generate stable phyllotaxis, and thus that phyllotaxis order requires a fully functional peripheral zone, via *VIP3* activity.

We also analyzed the expression pattern of *STM*, which is downregulated in incipient primordia (Long et al., 1996) and induced in organ boundaries (Landrein et al., 2015a); *AHP6*, which is induced in emerging organs (Besnard et al., 2014); and *CUC3*, which is more highly expressed in organ boundaries (Hibara et al., 2006). All three genes have been related to phyllotaxis indirectly through their gene expression pattern (Burian et al., 2015; Peaucelle et al., 2007; Besnard et al., 2014; Gallois et al., 2002). In *vip3*, *STM*, *AHP6* and *CUC3* reporters were expressed at the same location as in the WT, i.e. downregulated in incipient organs (*STM*), induced in boundaries (*STM*, *CUC3*) and induced in emerging organs (*AHP6*). However, their expression pattern was consistent with the abnormal spatial organ initiation pattern observed in *vip3* (Fig. 5B, Fig. S4B,C,E). Together with the fact that these factors spatially mark organs later than DR5, these results are consistent with a role of VIP3 in controlling the regularity of phyllotaxis early on during the specification of organ initiation sites, and through the auxin pathway rather than other pathways.

## DISCUSSION

The molecular mechanisms behind patterning in multicellular organisms are becoming unraveled in all species, from stomata and trichome patterning in plants to hairs and somites in animals (Oates et al., 2009). Here, we explored whether regulators of pattern variance exist, in parallel with pattern effectors. To do so, we focus on one of the most striking mathematical patterns in biology, phyllotaxis, the ordered pattern of organ initiation at the SAM, in *Arabidopsis*.

The mathematical precision of phyllotaxis has fascinated scientists for centuries. D'Arcy Thompson, in his chapter ‘On leaf-arrangement, or phyllotaxis’, quoting Nehemiah Grew, writes: ‘From the contemplation of plants, men might first be invited to mathematical enquirys’ (p. 912, Thompson, 1942). Consistently, most theoretical work on phyllotaxis emphasizes its regularity (e.g. Douady and Couder, 1992). Interestingly, finding mutants with irregular phyllotaxis has proven extremely difficult: mutants with irregular architecture, and thus possibly defects in phyllotaxis, turned out to have defects in stem growth (e.g. Landrein et al., 2013) or in plastochron, i.e. in the temporal sequence, but not in the initial position, of organ emergence (e.g. Besnard et al., 2014). Here, we identify the Paf1 complex as a regulator of phyllotactic regularity at the meristem. Our study thus demonstrates that variability in phyllotaxis can arise from internal noise due to genetic background (WT versus *vip3* mutants; Fig. 5C).

From the literature, evidence for a role of auxin in phyllotaxis regularity is indirect: it is well-established that auxin drives organ outgrowth very early on at the SAM (Besnard et al., 2011); yet, because the auxin-related mutants (*mp*, *pin1*, *pid*) do not produce organs, the role of auxin in phyllotaxis regularity could be questioned. Because auxin activity is disturbed in the *vip3* mutant, this work further consolidates auxin as a central player in phyllotaxis regularity, consistent with predictions from computational models and experimental work on organogenesis so far.

Our data also show that plant architecture can be influenced by environmental conditions (long days at 21°C versus continuous light at 16°C), confirming that growth conditions leading to more vigorous plants also increase the probability of affecting the temporal sequence of organ emergence (Landrein et al., 2015b), in parallel with phyllotaxis defects, at the shoot apex.

Paf1c might also contribute to the reproducibility of other morphogenetic events in animals (Akanuma et al., 2007; Nguyen et al., 2010; Langenbacher et al., 2011; Kubota et al., 2014). Previous studies have suggested that variability at the local scale marks cell identity (Singh et al., 2010; Laslo et al., 2006), primes organogenesis (Uyttewaal et al., 2012), contributes to reproducible organ shapes (Hong et al., 2016) and is actively maintained (Abley et al., 2016; Uyttewaal et al., 2012). Because the Paf1 complex has a rather pleiotropic biochemical role, from transcriptional regulation to the control of histone modification and mRNA stability, revisiting the role of Paf1c in relation to phenotypic variance could help us understand how genome-wide variability is buffered to generate reproducible shapes.

## MATERIALS AND METHODS

### Plant lines and growth conditions

All procedures were performed on plants from the Col-0 ecotype. The *pDR5::GFP* (Vernoux et al., 2011), *pSTM::CFP-N7* (Landrein et al., 2015a), *pCUC3::CFP* (Landrein et al., 2015a), *pAHFP6::GFP* (Besnard et al., 2014), *pMP:3xGFP* (Rademacher et al., 2011) reporter lines and T-DNA insertion lines *vip3-1* (salk139885), *vip3-2* (salk083364), *vip3-6* (Jensen et al., 2017) and *vip6* (salk065364) were used for this study (genotyping primers are listed in Table S1). In ‘long-day’ conditions, plants were continuously grown at 16 h/8 h light/dark period at 21°C. In ‘continuous-light’ conditions, plants were first grown for 3 weeks at 8 h/16 h light/dark at 21°C and then transferred to continuous light at 16°C.

### Phyllotaxis measurement

Internode distances were measured on fully elongated main stems, from the insertion site of the last secondary branch to the last silique. The measurement of divergence angles between the successive siliques, and the mathematical analysis of permutations in individual angle sequences was performed as described by Besnard et al. (2014).

### Meristem dissection

Stems were cut and the SAM was dissected when it switched to an inflorescence meristem identity, i.e. between the appearance of the first flower to the appearance of first silique (stages 13 to 17; Smyth et al., 1990) and transferred onto a half MS medium with vitamins and 0.125 µg/µl benzylaminopurine for imaging as described by Hamant et al. (2014).

### Confocal laser scanning microscopy and image analysis

Dissected meristems and plants grown *in vitro* were imaged with a water-dipping lens (×25, NA=0.8) using an SP8 confocal microscope (Leica, Germany) to generate a stack of optical sections with an interval of 0.25 µm between slices. The membranes were stained with FM4-64. Meristem size and divergence angles between successive primordia from the confocal stack images, as described by Besnard et al. (2014). Statistical analysis was performed using either Microsoft Excel or R software. Two-tailed Student's *t*-tests were performed to compare means of independent biological replicates.

### *In situ* hybridization

*In situ* hybridization on paraplast-embedded tissues was performed as described by Vernoux et al. (2011). Shoot apices were sectioned into 7-µm-thick slices. The probes for the coding regions of *VIP3*, *STM*, *CLV3*, *MP* and *GFP* were amplified with specific primers (listed in Table S1) using the GoTaq G2 Polymerase (Promega, 9PIM784). *In vitro* transcription and digoxigenin (DIG) labeling of the probes were performed using T7 RNA polymerase (Promega, P2077) and DIG RNA Labeling Mix (Roche, 11277073910).

## Supplementary Material

Supplementary information

Supplementary information

## References

[DEV154369C1] Abley K., Locke J. C. W. and Leyser H. M. O. (2016). Developmental mechanisms underlying variable, invariant and plastic phenotypes. *Ann. Bot.* 117, 733-748. 10.1093/aob/mcw016 27072645 PMC4845803

[DEV154369C2] Aida M., Vernoux T., Furutani M., Traas J. and Tasaka M. (2002). Roles of PIN-FORMED1 and MONOPTEROS in pattern formation of the apical region of the Arabidopsis embryo. *Development* 129, 3965-3974. 12163400 10.1242/dev.129.17.3965

[DEV154369C3] Akanuma T., Koshida S., Kawamura A., Kishimoto Y. and Takada S. (2007). Paf1 complex homologues are required for Notch-regulated transcription during somite segmentation. *EMBO Rep.* 8, 858-863. 10.1038/sj.embor.7401045 17721442 PMC1973952

[DEV154369C4] Bayer E. M., Smith R. S., Mandel T., Nakayama N., Sauer M., Prusinkiewicz P. and Kuhlemeier C. (2009). Integration of transport-based models for phyllotaxis and midvein formation. *Genes Dev.* 23, 373-384. 10.1101/gad.497009 19204121 PMC2648550

[DEV154369C5] Besnard F., Vernoux T. and Hamant O. (2011). Organogenesis from stem cells in planta: multiple feedback loops integrating molecular and mechanical signals. *Cell. Mol. Life Sci.* 68, 2885-2906. 10.1007/s00018-011-0732-4 21655916 PMC11115100

[DEV154369C6] Besnard F., Refahi Y., Morin V., Marteaux B., Brunoud G., Chambrier P., Rozier F., Mirabet V., Legrand J., Lainé S. et al. (2014). Cytokinin signalling inhibitory fields provide robustness to phyllotaxis. *Nature* 505, 417-421. 10.1038/nature12791 24336201

[DEV154369C7] Bhatia N., Bozorg B., Larsson A., Ohno C., Jönsson H. and Heisler M. G. (2016). Auxin acts through MONOPTEROS to regulate plant cell polarity and pattern phyllotaxis. *Curr. Biol.* 26, 3202-3208. 10.1016/j.cub.2016.09.044 27818174 PMC5154752

[DEV154369C8] Burian A., Raczyńska-Szajgin M., Borowska-Wykręt D., Piatek A., Aida M. and Kwiatkowska D. (2015). The CUP-SHAPED COTYLEDON2 and 3 genes have a post-meristematic effect on Arabidopsis thaliana phyllotaxis. *Ann. Bot.* 115, 807-820. 10.1093/aob/mcv013 25681504 PMC4373294

[DEV154369C9] Chu X., Qin X., Xu H., Li L., Wang Z., Li F., Xie X., Zhou H., Shen Y. and Long J. (2013). Structural insights into Paf1 complex assembly and histone binding. *Nucleic Acids Res.* 41, 10619-10629. 10.1093/nar/gkt819 24038468 PMC3905892

[DEV154369C10] Couder Y. (1998). Initial transitions, order and disorder in phyllotactic patterns: the ontogeny of Helanthus annuus. A case study. *Acta Soc. Bot. Pol.*, 67, 129-150.

[DEV154369C11] de Reuille P. B., Bohn-Courseau I., Ljung K., Morin H., Carraro N., Godin C. and Traas J. (2006). Computer simulations reveal properties of the cell-cell signaling network at the shoot apex in Arabidopsis. *Proc. Natl. Acad. Sci. USA* 103, 1627-1632. 10.1073/pnas.0510130103 16432202 PMC1360567

[DEV154369C12] Dermody J. L. and Buratowski S. (2010). Leo1 subunit of the yeast paf1 complex binds RNA and contributes to complex recruitment. *J. Biol. Chem.* 285, 33671-33679. 10.1074/jbc.M110.140764 20732871 PMC2962465

[DEV154369C13] Dorcey E., Rodriguez-Villalon A., Salinas P., Santuari L., Pradervand S., Harshman K. and Hardtke C. S. (2012). Context-dependent dual role of SKI8 homologs in mRNA synthesis and turnover. *PLoS Genet.* 8, e1002652 10.1371/journal.pgen.1002652 22511887 PMC3325215

[DEV154369C14] Douady S. and Couder Y. (1992). Phyllotaxis as a physical self-organized growth process. *Phys. Rev. Lett.* 68, 2098-2101. 10.1103/PhysRevLett.68.2098 10045303

[DEV154369C15] Fletcher J. C., Brand U., Running M. P., Simon R. and Meyerowitz E. M. (1999). Signaling of cell fate decisions by CLAVATA3 in Arabidopsis shoot meristems. *Science* 283, 1911-1914. 10.1126/science.283.5409.1911 10082464

[DEV154369C16] Gallois J.-L., Woodward C., Reddy G. V. and Sablowski R. (2002). Combined SHOOT MERISTEMLESS and WUSCHEL trigger ectopic organogenesis in Arabidopsis. *Development* 129, 3207-3217. 12070095 10.1242/dev.129.13.3207

[DEV154369C17] Gälweiler L., Guan C., Müller A., Wisman E., Mendgen K., Yephremov A. and Palme K. (1998). Regulation of polar auxin transport by AtPIN1 in Arabidopsis vascular tissue. *Science* 282, 2226-2230. 10.1126/science.282.5397.2226 9856939

[DEV154369C18] Giulini A., Wang J. and Jackson D. (2004). Control of phyllotaxy by the cytokinin-inducible response regulator homologue ABPHYL1. *Nature* 430, 1031-1034. 10.1038/nature02778 15329722

[DEV154369C19] Gruel J., Landrein B., Tarr P., Schuster C., Refahi Y., Sampathkumar A., Hamant O., Meyerowitz E. M. and Jönsson H. (2016). An epidermis-driven mechanism positions and scales stem cell niches in plants. *Sci. Adv.* 2, e1500989 10.1126/sciadv.1500989 27152324 PMC4846443

[DEV154369C20] Guédon Y., Refahi Y., Besnard F., Farcot E., Godin C. and Vernoux T. (2013). Pattern identification and characterization reveal permutations of organs as a key genetically controlled property of post-meristematic phyllotaxis. *J. Theor. Biol.* 338, 94-110. 10.1016/j.jtbi.2013.07.026 23948553

[DEV154369C21] Gupta P. B., Fillmore C. M., Jiang G., Shapira S. D., Tao K., Kuperwasser C. and Lander E. S. (2011). Stochastic state transitions give rise to phenotypic equilibrium in populations of cancer cells. *Cell* 146, 633-644. 10.1016/j.cell.2011.07.026 21854987

[DEV154369C22] Hamant O., Das P. and Burian A. (2014). Time-lapse imaging of developing meristems using confocal laser scanning microscope. *Methods Mol. Biol. Clifton NJ* 1080, 111-119. 10.1007/978-1-62703-643-6_9 24132423

[DEV154369C23] He Y., Doyle M. R. and Amasino R. M. (2004). PAF1-complex-mediated histone methylation of FLOWERING LOCUS C chromatin is required for the vernalization-responsive, winter-annual habit in Arabidopsis. *Genes Dev.* 18, 2774-2784. 10.1101/gad.1244504 15520273 PMC528897

[DEV154369C24] Heisler M. G., Ohno C., Das P., Sieber P., Reddy G. V., Long J. A. and Meyerowitz E. M. (2005). Patterns of auxin transport and gene expression during primordium development revealed by live imaging of the Arabidopsis inflorescence meristem. *Curr. Biol.* 15, 1899-1911. 10.1016/j.cub.2005.09.052 16271866

[DEV154369C25] Heisler M. G., Hamant O., Krupinski P., Uyttewaal M., Ohno C., Jönsson H., Traas J. and Meyerowitz E. M. (2010). Alignment between PIN1 polarity and microtubule orientation in the shoot apical meristem reveals a tight coupling between morphogenesis and auxin transport. *PLoS Biol.* 8, e1000516 10.1371/journal.pbio.1000516 20976043 PMC2957402

[DEV154369C26] Hibara K., Karim M. R., Takada S., Taoka K., Furutani M., Aida M. and Tasaka M. (2006). Arabidopsis CUP-SHAPED COTYLEDON3 regulates postembryonic shoot meristem and organ boundary formation. *Plant Cell* 18, 2946-2957. 10.1105/tpc.106.045716 17122068 PMC1693926

[DEV154369C27] Hong L., Dumond M., Tsugawa S., Sapala A., Routier-Kierzkowska A.-L., Zhou Y., Chen C., Kiss A., Zhu M., Hamant O. et al. (2016). Variable cell growth yields reproducible organ development through spatiotemporal averaging. *Dev. Cell* 38, 15-32. 10.1016/j.devcel.2016.06.016 27404356

[DEV154369C28] Itoh J.-I., Kitano H., Matsuoka M. and Nagato Y. (2000). Shoot organization genes regulate shoot apical meristem organization and the pattern of leaf primordium initiation in rice. *Plant Cell* 12, 2161-2174. 10.1105/tpc.12.11.2161 11090216 PMC150165

[DEV154369C29] Jaehning J. A. (2010). The Paf1 complex: platform or player in RNA polymerase II transcription? *Biochim. Biophys. Acta* 1799, 379-388. 10.1016/j.bbagrm.2010.01.001 20060942 PMC2862274

[DEV154369C30] Jensen G. S., Fal K., Hamant O. and Haswell E. S. (2017). The RNA polymerase-associated factor 1 complex is required for plant touch responses. *J. Exp. Bot.* 68, 499-511. 10.1093/jxb/erw439 28204553 PMC5441907

[DEV154369C31] Kim S., Kim J.-D., Chung A.-Y., Kim H.-S., Kim Y.-S., Kim M.-J., Koun S., Lee Y. M., Rhee M., Park H.-C. et al. (2012). Antagonistic regulation of PAF1C and p-TEFb is required for oligodendrocyte differentiation. *J. Neurosci. Off. J. Soc. Neurosci.* 32, 8201-8207. 10.1523/JNEUROSCI.5344-11.2012 PMC6703655 22699901

[DEV154369C32] Koch C., Wollmann P., Dahl M. and Lottspeich F. (1999). A role for Ctr9p and Paf1p in the regulation of G1 cyclin expression in yeast. *Nucleic Acids Res.* 27, 2126-2134. 10.1093/nar/27.10.2126 10219085 PMC148432

[DEV154369C33] Kubota Y., Tsuyama K., Takabayashi Y., Haruta N., Maruyama R., Iida N. and Sugimoto A. (2014). The PAF1 complex is involved in embryonic epidermal morphogenesis in Caenorhabditis elegans. *Dev. Biol.* 391, 43-53. 10.1016/j.ydbio.2014.04.002 24721716

[DEV154369C34] Landrein B., Lathe R., Bringmann M., Vouillot C., Ivakov A., Boudaoud A., Persson S. and Hamant O. (2013). Impaired cellulose synthase guidance leads to stem torsion and twists phyllotactic patterns in Arabidopsis. *Curr. Biol. CB* 23, 895-900. 10.1016/j.cub.2013.04.013 23623553

[DEV154369C35] Landrein B., Kiss A., Sassi M., Chauvet A., Das P., Cortizo M., Laufs P., Takeda S., Aida M., Traas J. et al. (2015a). Mechanical stress contributes to the expression of the STM homeobox gene in Arabidopsis shoot meristems. *Elife* 4, e07811 10.7554/eLife.07811 26623515 PMC4666715

[DEV154369C36] Landrein B., Refahi Y., Besnard F., Hervieux N., Mirabet V., Boudaoud A., Vernoux T. and Hamant O. (2015b). Meristem size contributes to the robustness of phyllotaxis in Arabidopsis. *J. Exp. Bot.* 66, 1317-1324. 10.1093/jxb/eru482 25504644 PMC4339594

[DEV154369C37] Langenbacher A. D., Nguyen C. T., Cavanaugh A. M., Huang J., Lu F. and Chen J.-N. (2011). The PAF1 complex differentially regulates cardiomyocyte specification. *Dev. Biol.* 353, 19-28. 10.1016/j.ydbio.2011.02.011 21338598 PMC3075326

[DEV154369C38] Laslo P., Spooner C. J., Warmflash A., Lancki D. W., Lee H.-J., Sciammas R., Gantner B. N., Dinner A. R. and Singh H. (2006). Multilineage transcriptional priming and determination of alternate hematopoietic cell fates. *Cell* 126, 755-766. 10.1016/j.cell.2006.06.052 16923394

[DEV154369C39] Leyser H. M. O. and Furner I. J. (1992). Characterisation of three shoot apical meristem mutants of Arabidopsis thaliana. *Development* 116, 397-403.

[DEV154369C40] Long J. A., Moan E. I., Medford J. I. and Barton M. K. (1996). A member of the KNOTTED class of homeodomain proteins encoded by the STM gene of Arabidopsis. *Nature* 379, 66-69. 10.1038/379066a0 8538741

[DEV154369C41] Mirabet V., Besnard F., Vernoux T. and Boudaoud A. (2012). Noise and robustness in phyllotaxis. *PLoS Comput. Biol.* 8, e1002389 10.1371/journal.pcbi.1002389 22359496 PMC3280957

[DEV154369C42] Nguyen C. T., Langenbacher A., Hsieh M. and Chen J.-N. (2010). The PAF1 complex component Leo1 is essential for cardiac and neural crest development in zebrafish. *Dev. Biol.* 341, 167-175. 10.1016/j.ydbio.2010.02.020 20178782 PMC2854236

[DEV154369C43] Nordick K., Hoffman M. G., Betz J. L. and Jaehning J. A. (2008). Direct interactions between the Paf1 complex and a cleavage and polyadenylation factor are revealed by dissociation of Paf1 from RNA polymerase II. *Eukaryot. Cell* 7, 1158-1167. 10.1128/EC.00434-07 18469135 PMC2446681

[DEV154369C44] Oates A. C., Gorfinkiel N., González-Gaitán M. and Heisenberg C.-P. (2009). Quantitative approaches in developmental biology. *Nat. Rev. Genet.* 10, 517-530. 10.1038/nrg2548 19584811

[DEV154369C45] Oh S., Zhang H., Ludwig P. and van Nocker S. (2004). A mechanism related to the yeast transcriptional regulator Paf1c is required for expression of the Arabidopsis FLC/MAF MADS box gene family. *Plant Cell* 16, 2940-2953. 10.1105/tpc.104.026062 15472079 PMC527190

[DEV154369C46] Oh S., Park S. and van Nocker S. (2008). Genic and global functions for Paf1C in chromatin modification and gene expression in Arabidopsis. *PLoS Genet.* 4, e1000077 10.1371/journal.pgen.1000077 18725930 PMC2515192

[DEV154369C47] Park S., Oh S., Ek-Ramos J. and van Nocker S. (2010). PLANT HOMOLOGOUS TO PARAFIBROMIN is a component of the PAF1 complex and assists in regulating expression of genes within H3K27ME3-enriched chromatin. *Plant Physiol.* 153, 821-831. 10.1104/pp.110.155838 20363855 PMC2879801

[DEV154369C48] Peaucelle A., Morin H., Traas J. and Laufs P. (2007). Plants expressing a miR164-resistant CUC2 gene reveal the importance of post-meristematic maintenance of phyllotaxy in Arabidopsis. *Development* 134, 1045-1050. 10.1242/dev.02774 17251269

[DEV154369C49] Penheiter K. L., Washburn T. M., Porter S. E., Hoffman M. G. and Jaehning J. A. (2005). A posttranscriptional role for the yeast Paf1-RNA polymerase II complex is revealed by identification of primary targets. *Mol. Cell* 20, 213-223. 10.1016/j.molcel.2005.08.023 16246724

[DEV154369C50] Prasad K., Grigg S. P., Barkoulas M., Yadav R. K., Sanchez-Perez G. F., Pinon V., Blilou I., Hofhuis H., Dhonukshe P., Galinha C. et al. (2011). Arabidopsis PLETHORA transcription factors control phyllotaxis. *Curr. Biol.* 21, 1123-1128. 10.1016/j.cub.2011.05.009 21700457

[DEV154369C51] Rademacher E. H., Möller B., Lokerse A. S., Llavata-Peris C. I., van den Berg W. and Weijers D. (2011). A cellular expression map of the Arabidopsis AUXIN RESPONSE FACTOR gene family. *Plant J. Cell Mol. Biol.* 68, 597-606. 10.1111/j.1365-313X.2011.04710.x 21831209

[DEV154369C52] Refahi Y., Farcot E., Guédon Y., Besnard F., Vernoux T. and Godin C. (2011). A combinatorial model of phyllotaxis perturbations in Arabidopsis thaliana. *Comb. Pattern Matching*, 323-335. 10.1007/978-3-642-21458-5_28

[DEV154369C53] Refahi Y., Brunoud G., Farcot E., Jean-Marie A., Pulkkinen M., Vernoux T. and Godin C. (2016). A stochastic multicellular model identifies biological watermarks from disorders in self-organized patterns of phyllotaxis. *Elife* 5, e14093 10.7554/eLife.14093 27380805 PMC4947393

[DEV154369C54] Reinhardt D., Pesce E.-R., Stieger P., Mandel T., Baltensperger K., Bennett M., Traas J., Friml J. and Kuhlemeier C. (2003). Regulation of phyllotaxis by polar auxin transport. *Nature* 426, 255-260. 10.1038/nature02081 14628043

[DEV154369C55] Sadeghi L., Prasad P., Ekwall K., Cohen A. and Svensson J. P. (2015). The Paf1 complex factors Leo1 and Paf1 promote local histone turnover to modulate chromatin states in fission yeast. *EMBO Rep.* 16, 1673-1687. 10.15252/embr.201541214 26518661 PMC4687421

[DEV154369C56] Sahlin P., Söderberg B. and Jönsson H. (2009). Regulated transport as a mechanism for pattern generation: capabilities for phyllotaxis and beyond. *J. Theor. Biol.* 258, 60-70. 10.1016/j.jtbi.2009.01.019 19490869

[DEV154369C57] Sheldon K. E., Mauger D. M. and Arndt K. M. (2005). A Requirement for the Saccharomyces cerevisiae Paf1 complex in snoRNA 3′ end formation. *Mol. Cell* 20, 225-236. 10.1016/j.molcel.2005.08.026 16246725 PMC1839845

[DEV154369C58] Singh D. K., Ku C.-J., Wichaidit C., Steininger R. J., Wu L. F. and Altschuler S. J. (2010). Patterns of basal signaling heterogeneity can distinguish cellular populations with different drug sensitivities. *Mol. Syst. Biol.* 6, 369 10.1038/msb.2010.22 20461076 PMC2890326

[DEV154369C59] Smith R. S., Guyomarc'h S., Mandel T., Reinhardt D., Kuhlemeier C. and Prusinkiewicz P. (2006). A plausible model of phyllotaxis. *Proc. Natl. Acad. Sci. USA* 103, 1301-1306. 10.1073/pnas.0510457103 16432192 PMC1345713

[DEV154369C60] Smyth D. R., Bowman J. L. and Meyerowitz E. M. (1990). Early flower development in Arabidopsis. *Plant Cell* 2, 755-767. 10.1105/tpc.2.8.755 2152125 PMC159928

[DEV154369C61] Stoma S., Lucas M., Chopard J., Schaedel M., Traas J. and Godin C. (2008). Flux-based transport enhancement as a plausible unifying mechanism for auxin transport in meristem development. *PLoS Comput. Biol.* 4, e1000207 10.1371/journal.pcbi.1000207 18974825 PMC2565506

[DEV154369C62] Szczesny T., Routier-Kierzkowska A.-L. and Kwiatkowska D. (2009). Influence of clavata3-2 mutation on early flower development in Arabidopsis thaliana: quantitative analysis of changing geometry. *J. Exp. Bot.* 60, 679-695. 10.1093/jxb/ern312 19088334 PMC2651453

[DEV154369C63] Takagi N. and Ueguchi C. (2012). Enhancement of meristem formation by bouquet-1, a mis-sense allele of the vernalization independence 3 gene encoding a WD40 repeat protein in Arabidopsis thaliana. *Genes Cells Devoted Mol. Cell. Mech.* 17, 982-993. 10.1111/gtc.12014 23134555

[DEV154369C64] Thompson D. W. (1942). *On Growth and Form*, 2nd edn Cambridge, UK: Cambridge University Press.

[DEV154369C65] Ulmasov T., Murfett J., Hagen G. and Guilfoyle T. J. (1997). Aux/IAA proteins repress expression of reporter genes containing natural and highly active synthetic auxin response elements. *Plant Cell* 9, 1963-1971. 10.1105/tpc.9.11.1963 9401121 PMC157050

[DEV154369C66] Uyttewaal M., Burian A., Alim K., Landrein B., Borowska-Wykręt D., Dedieu A., Peaucelle A., Ludynia M., Traas J., Boudaoud A. et al. (2012). Mechanical stress acts via katanin to amplify differences in growth rate between adjacent cells in Arabidopsis. *Cell* 149, 439-451. 10.1016/j.cell.2012.02.048 22500806

[DEV154369C67] Vernoux T., Brunoud G., Farcot E., Morin V., Van den Daele H., Legrand J., Oliva M., Das P., Larrieu A., Wells D. et al. (2011). The auxin signalling network translates dynamic input into robust patterning at the shoot apex. *Mol. Syst. Biol.* 7, 508 10.1038/msb.2011.39 21734647 PMC3167386

[DEV154369C68] Wernet M. F., Mazzoni E. O., Çelik A., Duncan D. M., Duncan I. and Desplan C. (2006). Stochastic spineless expression creates the retinal mosaic for colour vision. *Nature* 440, 174-180. 10.1038/nature04615 16525464 PMC3826883

[DEV154369C69] Zhang H. and van Nocker S. (2002). The VERNALIZATION INDEPENDENCE 4 gene encodes a novel regulator of FLOWERING LOCUS C. *Plant J. Cell Mol. Biol.* 31, 663-673. 10.1046/j.1365-313X.2002.01380.x 12207655

[DEV154369C70] Zhang H., Ransom C., Ludwig P. and van Nocker S. (2003). Genetic analysis of early flowering mutants in Arabidopsis defines a class of pleiotropic developmental regulator required for expression of the flowering-time switch flowering locus C. *Genetics* 164, 347-358. 12750345 10.1093/genetics/164.1.347 PMC1462564

[DEV154369C71] Zhang K., Haversat J. M. and Mager J. (2013). CTR9/PAF1c regulates molecular lineage identity, histone H3K36 trimethylation and genomic imprinting during preimplantation development. *Dev. Biol.* 383, 15-27. 10.1016/j.ydbio.2013.09.005 24036311 PMC4903072

