# Supplementary material for: Phyllotactic regularity requires the Paf1 complex in Arabidopsis
Source: Development. 2017 Dec 1;144(23):4428–36. doi: 10.1242/dev.154369 (PMC5769633; doi:10.1242/dev.154369)
Supplement: Supplementary information [file develop-144-154369-s1.pdf]

Figure S1

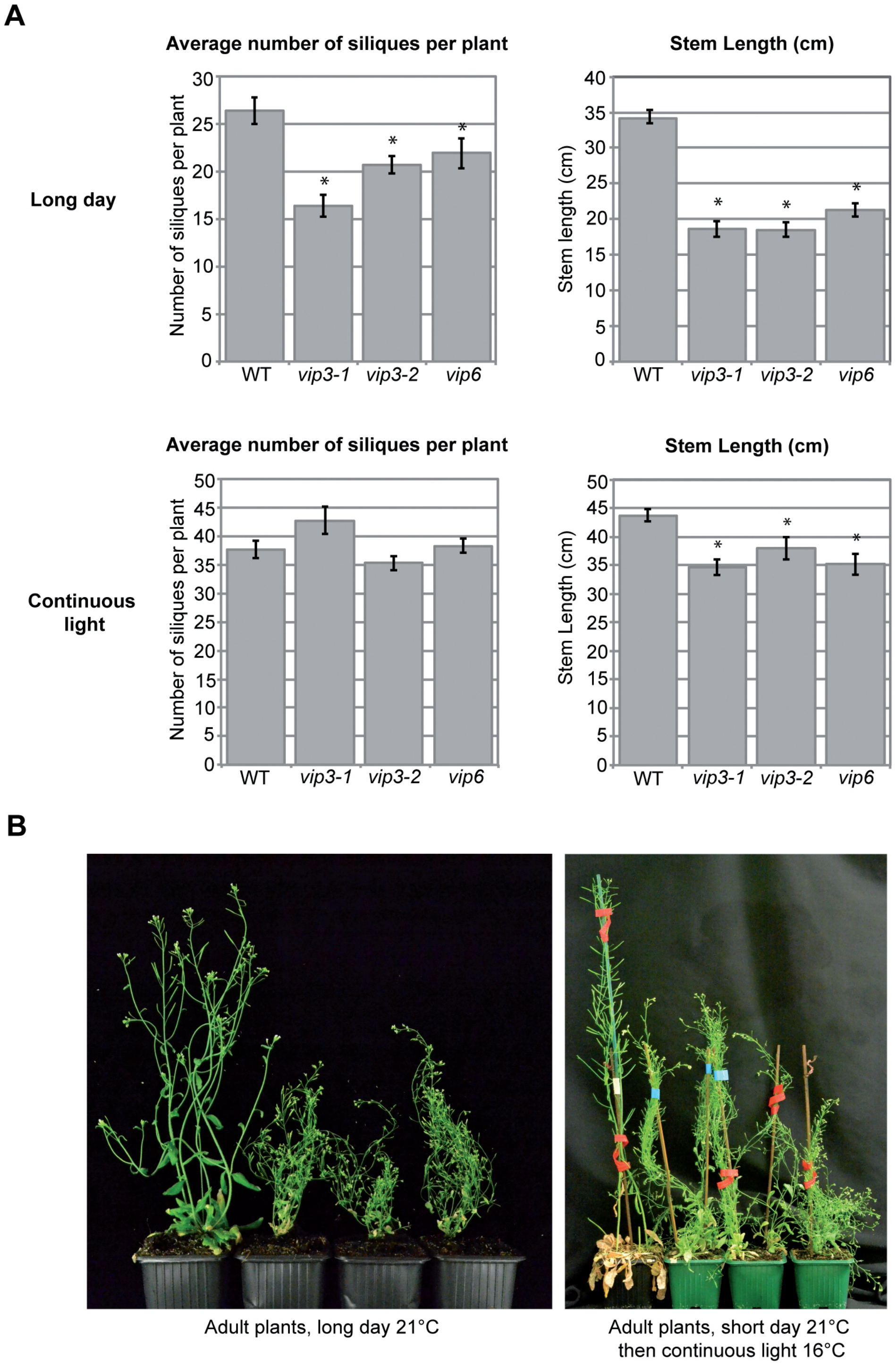

Fig. S1. Effect of growth conditions on *vip* mutant phenotype.

(A) Inflorescence stem length (right) and number of siliques along the main inflorescence stems (left) from plants grown at long day 21°C (referred as "long day") and short day 21°C then continuous light 16°C (referred as "continuous light"). Sample size: number of siliques, long day: WT (n=20), *vip3-1* (n=15), *vip3-2* (n=15), and *vip6* (n=18)); number of silique, continuous light: WT (n=54), *vip3-1* (n=20), *vip3-2* (n=52), and *vip6* (n=30); stem length, long day: WT (n=19), *vip3-1* (n=15), *vip3-2* (n=22), *vip6* (n=14); stem length, continuous light: WT (n=22), *vip3-2* (n=18), *vip3-1* (n=18), *vip6* (n=12). The error bars represent the Standard error of mean, the results were considered significant when α≤0.05% by two-tailed Student tests.
(B) WT and *vip* mutant phenotypes of adult plants grown in long day (left) and continuous light (right) conditions.

Development • Supplementary information

Figure S2

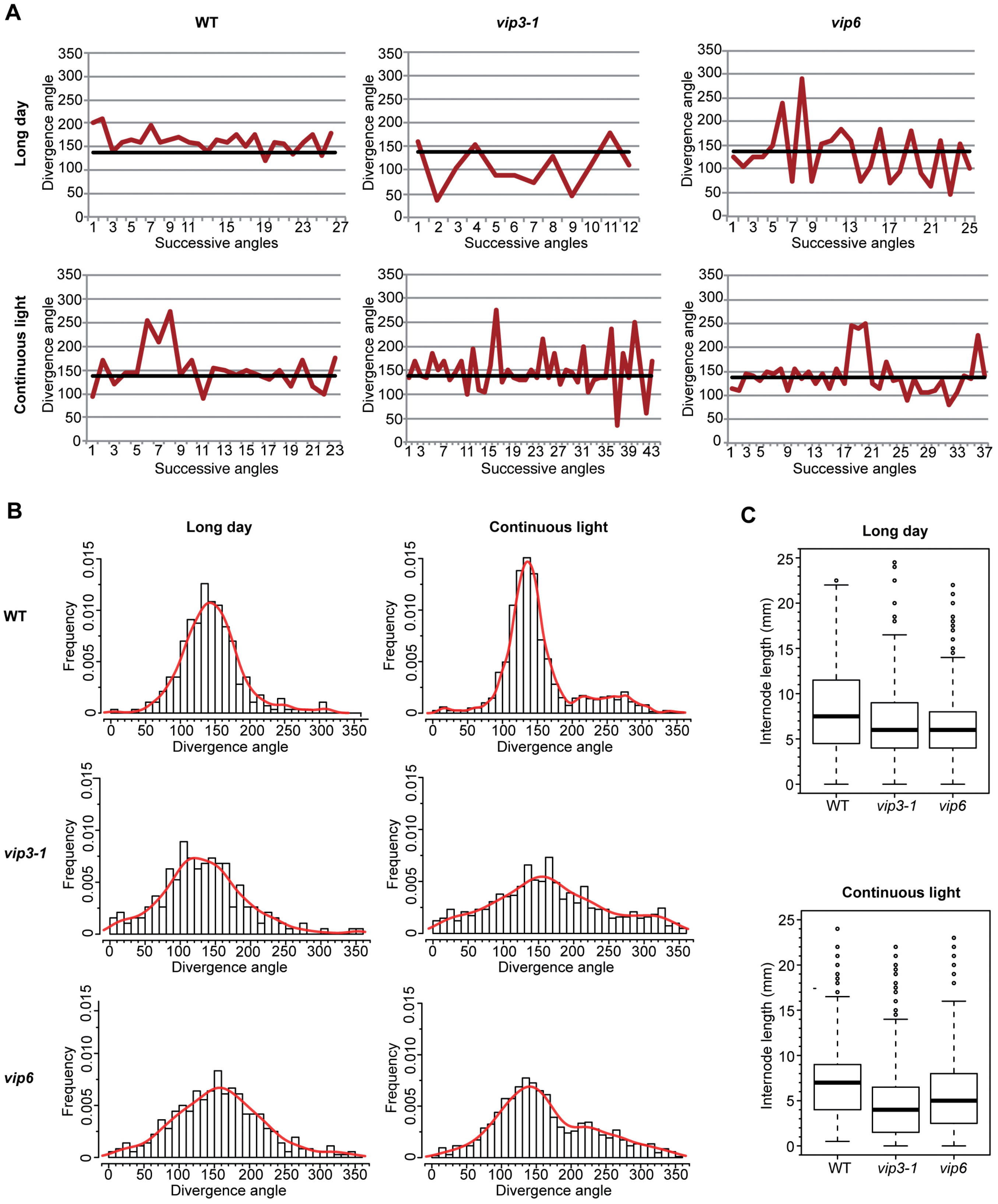

Fig. S2. Phyllotaxis is affected in *vip3* and *vip6* mutant plants.

(A) Sequence of divergence angles in representative WT, *vip3-1* and *vip6* plants grown in long day (upper panel) or continuous light (lower panel) conditions. The thick black line on each graph represents the canonical angle of 137°. (B) Frequency of divergence angles between successive siliques in WT, *vip3-1* and *vip6* plants grown in long day (left panel, WT: 305 angles, 20 plants; *vip3-1*: 201 angle, 15 plants; *vip6*: 366 angles, 18 plants) and continuous light (right panel, WT: 1997 angles, 54 plants, *vip3-1*: 820 angles, 20 plants, *vip6*: 1046 angles, 30 plants) conditions. (C) Average internode length on the inflorescence stem in WT, *vip3-1* and *vip6* plants grown at long day (left panel, WT: 520 internodes, 20 plants; *vip3-1*: 246 internodes, 15 plants; *vip6*: 420 internodes, 18 plants) and continuous light (right panel, WT: 620 internodes, 21 plants; *vip3-1*: 939 internodes, 20 plants; vip6: 1082 internodes, 30 plants) conditions. The black line in the boxplot represents the median, the box represents the distribution range of 50% of the measured values and the bars (whiskers) illustrate the upper and lower quartiles. The average internode length on *vip3* mutant plants is not significantly different from that of the wild type, but the frequency of shorter internodes is increased when compared to wild-type stems

Development • Supplementary information

Figure S3

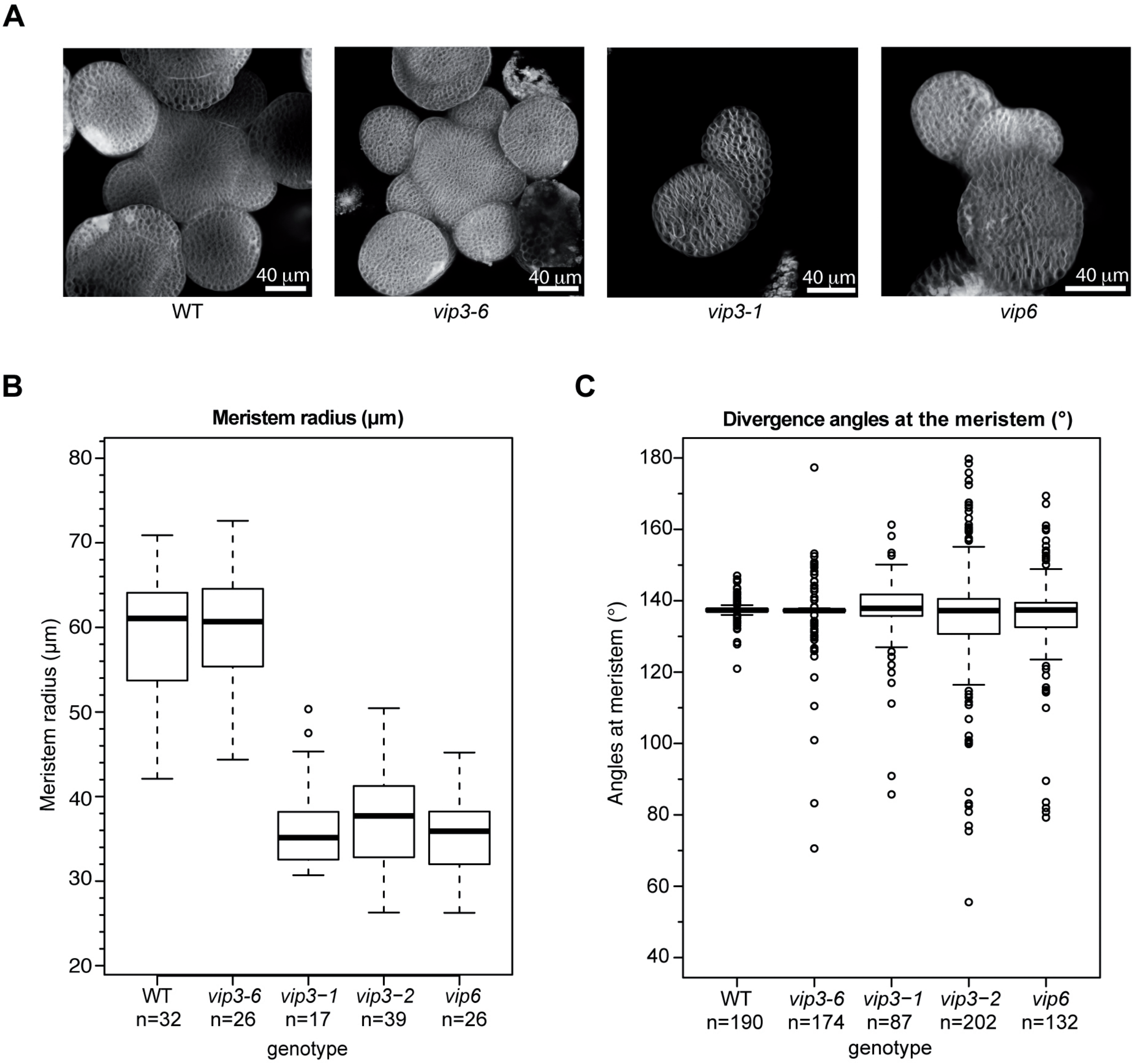

Fig. S3. Meristem size and divergence angles in WT, *vip3-1*, *vip3-6* and *vip6*.

(A) Representative WT, *vip3-6*, *vip3-1*, *vip6* meristems (surface projections), labelled with FM4-64. (B) Meristem radius (µm) in WT (n=32), *vip3-6* (n=26), *vip3-1* (n=17), *vip3-2* (n=39) and *vip6* (n=26) grown in continuous light conditions. Data for the WT and *vip3-2* are reproduced from Figure 4 to ease the comparison between genotypes. Meristem radius was measured as described in (Landrein et al., 2015b) ; $\alpha \leq 0.05\%$ by two-tailed Student tests. (C) Divergence angle between primordia at the SAM in WT (n=190), *vip3-6* (n=172), *vip3-1* (n=87), *vip3-2* (n=202) and *vip6* (n=132). The angles between the successive organs were estimated as described in (Landrein et al., 2015b).

Figure S4

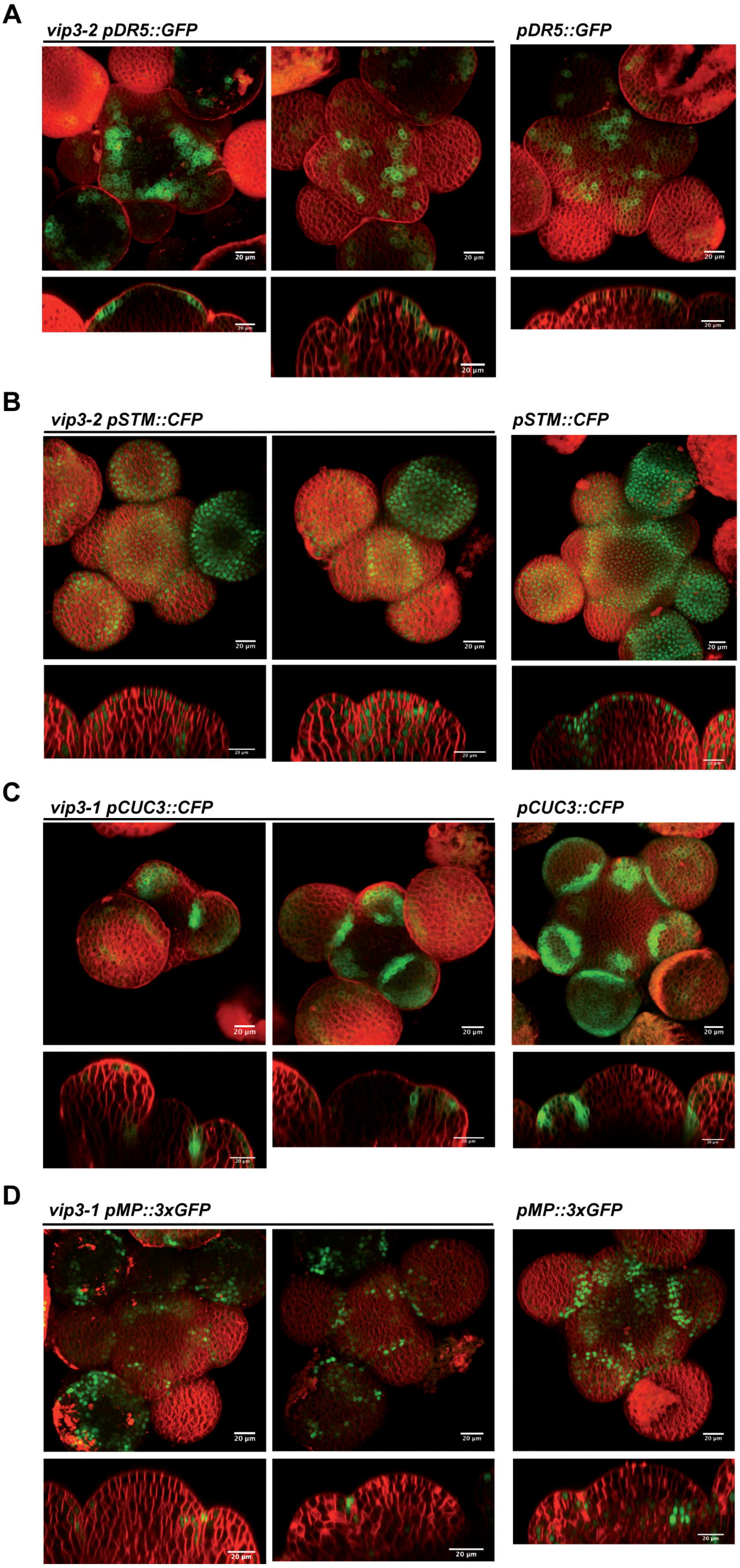

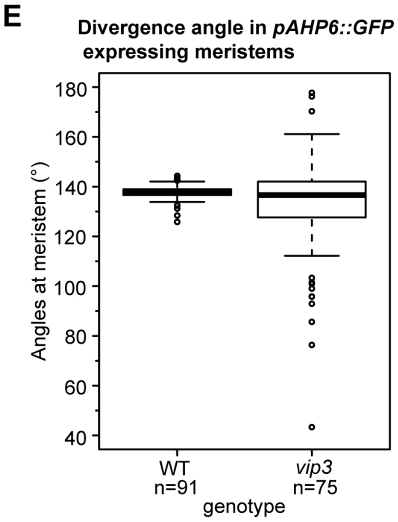

Fig. S4. *pDR5::GFP*, *pSTM::CFP-N7*, *pCUC3::CFP* and *pMP::3xGFP* expression patterns in *vip3*

(A) Two examples of *vip3-2* mutant meristems expressing *pDR5::GFP* (green), labelled with FM4-64 (red). Note the absence of focused DR5 peaks, when compared to the WT (Fig. 5A). Scale bars=20 µm. Lower panels: orthogonal optical sections of the same meristems.
(B) Two examples of *vip3-2* mutant meristems expressing *pSTM::CFP-N7* (green), labelled with FM4-64 (red). Note the stronger expression in boundaries in both WT and mutant. Scale bars=20 µm. Lower panels: orthogonal optical sections of the same meristems.
(C) Two examples of *vip3-1* mutant meristems expressing *pCUC3::CFP* (green), labelled with FM4-64 (red). Note the stronger expression in boundaries in both WT and mutant. Scale bars=20 µm. Lower panels: orthogonal optical sections of the same meristems.
(D) Two examples of *vip3-1* mutant meristems expressing *pMP::3xGFP* (green), labelled with FM4-64 (red). Note the weaker signal intensity when compared to the WT. Scale bars=20 µm. Lower panels: orthogonal optical sections of the same meristems.
(E) Divergence angles between successive primordia in WT (n=91) and *vip3-2* (n=75) mutant meristems with *pAHP6::GFP*. The angles were measured as described in (Landrein et al., 2015b). The boxplots represent the median and the quartile range distribution of the measured values.

Table S1. List of primers

**Name**	**Sequence**
*VIP3_in_situ*_F	GAGAATGAAACTCGCAGGTC
*VIP3_in_situ*_RT7	TAATACGACT CACTATAGGGCTTGTCATCA GAGACACTAGC
*STM_in_situ*_F	GAGATGTGATCCATTGGGAAAGG
*STM_in_situ*_RT7	TGTAATACGACTCACTATAGGGCGGTCCGATGTGTCCTATGATGATGATG
*CLV3_in_situ*_F	ATGTCCGGTCCAGTTCAACAAC
*CLV3_in_situ*_RT7	TGTAATACGACTCACTATAGGGCGGTCAGGTCCCGAAGGAACA
*MP_in_situ*_F	ATGATGGCTTCATTGTCTTGTG
*MP_in_situ*_RT7	TGTAATACGACTCACTATAGGGCTTATGAAACAGAAGTCTTAA
*GFP_in_situ*_F	AAGAACTTTTCACTGGAGTTGTCCC
*GFP_in_situ*_RT7	TGTAATACGACTCACTATAGGGCCGCTTCCATCTTCAATGTTGTGTCT
LBb1.3	ATTTTGCCGATTTCGGAAC
vip6 F	GATGCAACTGATGGGAAGGACTC
vip6 R	CACCCATACATCAGGCATCTGAAG
vip3-1 F	GACTGCAAGTACCACTTTCGC
vip3-1 R	TAATGGGAAACGACTTGCTTG
vip3-2 F	CTGACTGGATCTCTTGACGAGACG
vip3-2 R	GATACTCAGCAATTCCATATAGTACCCAAGC
vip3-6 F	GCAATTAGCTGACGACGGCGGG
vip3-6 R	GATCCAGCTCGTCCGGTCGCC

Development • Supplementary information
